# Termite Vibration Sensing: The Chordotonal Organs and Their Appendages

**DOI:** 10.1002/ece3.72287

**Published:** 2025-10-17

**Authors:** Travers M. Sansom, Joseph C. S. Lai, Benjamin J. Halkon, Theodore A. Evans, Sebastian Oberst

**Affiliations:** ^1^ Centre for Audio, Acoustics and Vibration University of Technology Sydney Sydney New South Wales Australia; ^2^ School of Engineering and Technology University of New South Wales Canberra Campbell Australian Capital Territory Australia; ^3^ School of Biological Sciences The University of Western Australia Crawley Western Australia Australia

**Keywords:** biotremology, Isoptera, Johnston's organ, signal amplification, subgenual organ, substrate‐borne vibration

## Abstract

Eusocial insects like termites and ants use diverse communication methods, including pheromones, sound, and vibrations. Termites, blind and with fewer glands, rely heavily on vibrations for foraging, communication, and predator avoidance. Their appendages detect subtle signals amid noise, though the underlying physiological mechanisms remain largely unknown and understudied. We explore the role of termite legs and antennae as sensory probes. These appendages receive the vibration signals, which are detected by the leg's subgenual organ and the Johnston's organ in the antenna, and which in turn convert these mechanical environmental signals into nerve impulses sent to the nervous system. We compare these appendages in termites and ants, two eusocial, subterranean insect groups that share ecological traits but differ in trophic roles, with ants being major predators of termites. Termite legs and antennae have lower slenderness ratios (legs: 19–35 vs. 48; antennae: 23–32 vs. 61). Wasps and bees fall in between. Assuming similar material properties, termite legs likely have lower stiffness and higher natural frequencies, enhancing vibration sensitivity. The subgenual organ's position near the head may further improve detection. These morphological traits suggest termites could be better adapted for sensing a broader range of vibrations than ants. However, more specimens and species of Isoptera and Formica need to be tested to validate this claim fully. Comparing the legs of termites with ants, we found that termite tibiae amplify lower‐frequency vibrations (~0–2.25 kHz), while ants show an amplification at higher frequencies (1.9–3.1 kHz). This suggests the vibrational sensitivity of termites is better adapted to wood‐borne signals, which corresponds to their food, whereas ants, as generalist foragers, are tuned for diverse terrains, including light structures, such as twigs, leaves, and other plant matter. Considered together, our findings suggest that termite legs may function as an integrated auditory complex.

## Graphical abstract

This study investigates how termites use their legs and antennae as vibration‐sensitive sensory organs, comparing them to ants, their eusocial predators. Termite appendages are morphologically adapted to detect lower‐frequency, wood‐borne vibrations, enhancing their foraging and predator avoidance abilities. These findings suggest that termite legs may function as an integrated auditory system, finely tuned to their ecological niche.

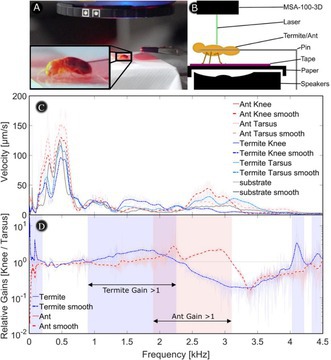

## 1 Background

Information gathering in social insects operates through multiple modalities, including chemical signals (pheromones), vision, airborne sound, substrate‐borne vibrations (biotremology), and tactile interaction, often functioning in an integrated manner (Hölldobler 1999). While termites (Isoptera), closely related to cockroaches (Blattodea), have a relatively simple and limited pheromone system, which consists of just 11 pheromone glands, far fewer than ants (39), wasps (14), or bees (21) (Vander Meer et al. 1999; Costa‐Leonardo et al. 2009; Matsuura 2012; Fu et al. 2020), studies show that they are also sensitive to volatile organic compounds (VOCs). VOCs are produced by plants, fungi, and soil microbes, which can act as attractants or repellents. Wood‐decaying fungi release terpenoids and alcohols that can either attract termites by signalling decayed, easily digestible wood or repel them by indicating harmful microbial activity (Boulogne et al. 2012; Rosengaus et al. 2011). Plant‐derived allelochemicals, such as essential oils rich in terpenes, are known to repel or even kill termites, and have been studied as biocontrol agents and are found in defence pheromones, which are often monoterpenes (α‐terpine), see Mitaka and Akino (2021). Also, soil microbes emit semiochemicals that may influence termite foraging and tunnelling behaviour, providing cues about resource quality or microbial hazards (Chouvenc et al. 2011). Thus, semiochemicals from the environment form a crucial part of how termites interact with their ecological niche, shaping their feeding, tunnelling, and defensive responses, yet vibrational signals also seem to play an essential part in nestmate communication, alarm, and coordination within colonies, and studies show that termites use the mode of substrate‐borne vibrations more than other social insects do (Hölldobler 1999; Hertel et al. 2011; Hager and Kirchner 2013; Sillam‐Dussès et al. 2023).

The importance of vibration signals to termites is well understood. Termites can detect and avoid interspecific competitors or predators based on mechanical vibrations caused by footstep vibrations (Oberst et al. 2017). Furthermore, they use vibrations produced from feeding to assess the quantity and quality of food (Evans et al. 2005; Inta et al. 2007; Oberst et al. 2018). They use clay to support load‐bearing structures (Oberst et al. 2016) and they manipulate moisture in timber to control the buckling stability of the wood under load (Oberst, Lenz, et al. 2019), both to access a greater proportion of the available food. Termites produce vibrations due to foraging activities that cause acoustic emissions and generate food‐source and species‐specific feeding signals (Evans et al. 2005; Inta et al. 2007; Oberst et al. 2018). Further, termites alert their colony members of threats using vibrations as ‘alarm signals’ (A. M. Stuart 1963). Threats can take the form of predators, primarily ants (Connétable et al. 1999; Hill et al. 2019; Oberst et al. 2017; Oberst, Lai, and Evans 2019; Tuma et al. 2020), or mammals such as anteaters and echidnas (McNab 1984; Redford 1987; Morton and James 1988; Bourguignon et al. 2017). Alarm signals are mostly produced by soldiers by drumming their heads against both the ceiling and the floor, generating a series of pulses (A. M. Stuart 1963, 1969; Röhrig et al. 1999; Hager and Kirchner 2014; van der Zee 2020), jerking their bodies anteriorly and posteriorly (Hertel et al. 2011; Hager et al. 2019), or combining drumming and jerking (Howse 1964a; Kirchner et al. 1994; Leis et al. 1994; Fink et al. 2006). The drumming alarm signal produced by termites has been found to resemble that of the footsteps of ants running across a veneer (Oberst et al. 2017). In body jerking, the substrate transmits the vibrations efficiently to close by, and the excitation source surrounding termites (Hertel et al. 2011; Hager et al. 2019).

Termite responses to drumming have been used for determining behavioural thresholds as a clear visual reaction can be observed (Hager and Kirchner 2014). The typical response of drumming consists of attracting soldiers to the area while repelling workers (Howse 1964a, 1964b; Leis et al. 1994). Hager and Kirchner (2014) expanded this approach, finding that termites can determine the direction of vibration based on the time delays of the wave reaching each leg. A recent study published by Sillam‐Dussès et al. (2023) explores the alarm signals of 15 species, and finds that alarm signals evolved before sociality; such signals have become increasingly complex in higher termites (Neoisoptera), and vibration is often combined with chemical communication.

The two vibration‐detecting organs are the subgenual organ (SGO) and the Johnston organ (JO). The function and physiology of the SGO have been studied by Howse (Howse 1964a, 1964b) and Wikantyoso et al. (2022), and in greater detail using micro‐computed tomography by Sansom et al. (2022). The JO may be used for vibration sensing also (Hunt and Richard 2013), but there are no studies on function and physiology in termites. In addition, little is known about the morphology and adaptations of vibration‐sensing organs, the effects of leg or antennae morphology on dynamic properties, and the function of these appendages as sensory probes.

Ants sense vibrations as well, yet seem to be less specialized in this modality (Buehlmann et al. 2012; Oberst et al. 2014). Ants can detect vibrations (e.g., Roces and Tautz 2001; Hager et al. 2017), but they rely more on their excellent vision and diverse semiochemicals, as is typical for all Hymenoptera. Ants have large compound eyes and colour vision; most species are diurnal, yet even nocturnal species use vision. Ants use semiochemicals (incl. pheromones as intraspecific semiochemicals) for colony identity, caste identity, laying trails, and communicating alarm (Hölldobler and Wilson 1990; Leonhardt et al. 2016). An exception is leaf cutter ants (*Atta* spp.), which make use of sensed vibrations and acoustics exciting the plant matter substrates they move on (twigs, leaves), and which allow them to locate sound source locations perceived through the time delay of waves using their legs as a sensor (Hager et al. 2017).

This study examines the morphology and function of two primary vibration‐sensing probes, legs and antennae, as well as key sensory organs, the subgenual organ (SGO) and Johnston's organ (JO), in termites. It does so in the context of biotremology, the study of how substrate‐borne vibrations are produced, transmitted, and detected for communication (Oster and Wilson 1978; Cocroft and Rodríguez 2005; Hunt and Richard 2013; Hill and Wessel 2016; Mortimer 2017). We review the literature and provide preliminary experimental results. We identify the critical information needed to understand vibrational communication in termites and explore how it can be obtained through experimental measurements and numerical simulations. Given that termites (*Isoptera*) are prey for ants, these groups exist in a predator–prey dynamic (Tuma et al. 2020), and that these groups appear to vary in their specialized vibration detection, we hypothesize they will display significant differences in their vibration‐sensing anatomy. Here, the main study species were the termite *Nasutitermes exitiosus* and the ant *Iridomyrmex purpureus* , with additional information from the termite *Coptotermes acinaciformis*, the honeybee *Apis mellifera*, and the wasp *Vespula germanica*.

## 2 Methodology

We identified from key databases (UTS Library, Scopus, and Google Scholar) published literature on vibration generation and sensing by termites and ants using the terms (“substrate‐borne vibration” OR “subgenual organ” OR “Johnston's organ”) AND/OR (Isoptera OR termites OR ant OR Hymenoptera) and identified about 132 articles. We extracted information about the characteristics of signals, legs, antennae, and the SGO and JO as sensing organs from these and complemented findings using measurements.

We collected samples of the termites *Na. exitiosus* and *C. lacteus* (Isoptera), and the ants *Ir. purpureus*, honeybees ( *A. mellifera* ), and wasps (*V. germanica*) from sites around Canberra, Australian Capital Territory, Australia (35.30° S, 149.17° E). *M. darwiniensis* was collected from Darwin, NT, Australia (12.46° S, 130.84° E). *A. mellifera* and *V. germanica* are examples of eusocial insects and serve as further comparisons to ants and termites, which are the focus of this study (Data S1).

We measured body length (measured from the mandibles to the end of the abdomen), leg length, antenna length, and thickness of the insects. The measurements were made from photographs taken with a 60 MP high‐resolution camera (Sony A7R V “9504 × 6336 pixels”) equipped with a macro lens (Sony FE 90 mm f/2.8 Macro G OSS set to a 1:1 magnification ratio of the full‐frame sensor 36 × 24 mm) with the insects on a slide of known size to determine the pixel length in mm (Data S2 and S3).

We conducted physical measurements via a Polytec MSA100‐3D Micro System Analyser to extract the vibration response spectrum to random vibrations of an ant's and termite legs and related this to their morphological features, especially length and thickness of appendages relative to body size. The MSA laser intensity/power was held at 30% (~250 μW) so that the tissue of the termite structure was not damaged (following Sansom et al. 2022). Measurements were smoothed by using a moving average filter of 100 samples (25 Hz), cf. Data S3 and S5.

Additional micro‐CT scans of the termite *Mastotermes darwiniensis* soldiers and workers and *Ir. purpureus* work were performed using the methodology and procedures outlined in Sansom et al. (2022) to measure the SGO leg angle (details of which can be found as Data S6).

## 3 Termite Vibration Sensing

### 3.1 Vibration Signals Emitted by Termites

Alarm signals have received the most attention in termites, particularly the stimuli that elicit an alarm signal (Table 1). Air currents, which can penetrate deep into the nest (Theraulaz et al. 1998), were most effective in 81% of the species studied, followed by vibrations (75%) and light (50%). Even though termites are blind, light, which they sense through photoreceptors in their cuticle and extraocular photoreception mechanism, has also been found to have a significant alarm response, attracting soldiers to the light edge and repelling workers (Park and Raina 2005). These stimuli indicate to the colony that the nest's protective barrier may have been breached by predators (Sweeney 1956; Howse 1966; Lubin and Montgomery 1981; Mahmood et al. 2020).

**TABLE 1:** Published information on stimuli and response alarm signals for termites found in laboratory experiments.

Family species	Stimulus			Response			Pulse repetition rate (Hz)		References
	Air	Light	Vib	Drum	S	W	Drum	Jerk	
Archotermopsidae									
*Zootermopsis angusticollis*	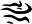	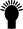	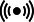	X	X	X	24	—	Howse (1964b); Meusemann et al. (2020)
*Z. nevadensis*	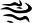	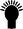	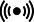	X	X	X	20	—	Meusemann et al. (2020); Kirchner et al. (1994)
Coptotermitinae									
*Coptotermes niger*	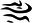			—	—	—	—	—	Meusemann et al. (2020); Bourguignon et al. (2015)
*Co. gestroi*	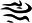	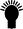	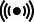	X	X	—	15.7	15.6	Meusemann et al. (2020); Bourguignon et al. (2015)
*Co. formosanus*				X	—	—	14	14	Meusemann et al. (2020); Bourguignon et al. (2015); Fink et al. (2006)
*Co. acinaciformis*				X	—	—	13	—	Meusemann et al. (2020); Bourguignon et al. (2015); Inta et al. (2009); Oberst et al. (2017)
Heterotermitinae									
*Reticulitermes santonensis*	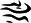		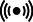	—	—	—	—	—	Meusemann et al. (2020); Bourguignon et al. (2015); Polizzi and Forschler (1998)
Kalotermitidae									
*Incisitermes marginipennis*	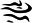	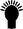	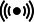	—	—	—	—	3.8	Meusemann et al. (2020); Bourguignon et al. (2015); Hager and Kirchner (2013); Bell et al. (2007)
Mastotermitidae									
*Mastotermes darwiniensis*	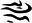		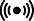	X	—	—	21	—	Meusemann et al. (2020); Bourguignon et al. (2015); Bell et al. (2007); Connétable et al. (1999); Delattre et al. (2015)
Termitidae									
*Pseudacanthotermes militaris*	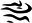	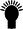	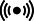	X	X	—	19	—	Bourguignon et al. (2015); Connétable et al. (1999)
*Macrotermes subhyalinus*	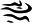		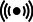	X	—	—	13	—	Meusemann et al. (2020); Bourguignon et al. (2015); Röhrig et al. (1999)
*Mac. natalensis*	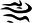	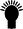	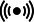	X	X	—	11	—	Meusemann et al. (2020); Bourguignon et al. (2015); Hager and Kirchner (2013)
*Mac. bellicosus*	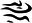		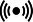	X	—	—	26	—	Meusemann et al. (2020); Bourguignon et al. (2015); Connétable et al. (1999)
*P. spiniger*	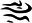	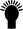	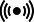	X	X	—	14	—	Kirchner et al. (1994); Connétable et al. (1999); Röhrig et al. (1999)
*Odontotermes sp*	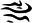	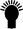	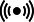	X	X	—	19	—	Hager and Kirchner (2013)
*Constrictotermes cyphergaster*				X	—	—	19	—	Bourguignon et al. (2015); Cristaldo et al. (2015)

*Note:* For the column ‘Stimulus’ we use the symbols 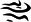 = air movement; 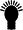 = light; and 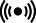 = vibration. For Response, Drum = species drums, but caste is unknown; S = soldier; W = worker drums. For Pulse repetition rate, Drum = Pulse repetition rate of drumming signal; Jerk = Pulse repetition rate of jerking signal. In the table, X or a specific value indicates some information is available; ‘—’ = no information found.

Vibration signals vary in their effectiveness in eliciting a response. However, since termites communicate via vibrations (Howse 1965a; Kirchner et al. 1994; Evans et al. 2009), it is possible that they can differentiate these vibrations from signals of concern, such as indicators of predation or alarm (Oberst et al. 2017). Playback of recorded natural alarm signals is effective in eliciting an alarm response (Hager and Kirchner 2014), as the signal's vibration signature likely becomes important (Oberst, Lenz, et al. 2019). These results show that air and light can be less specific in character, whereas vibration stimuli may require specific biologically relevant qualities and situational contexts (Howse 1964b; Castellanos and Barbosa 2006; Oberst et al. 2017; Oberst, Lenz, et al. 2019; Takanashi et al. 2019).

The pulse repetition rate of the alarm drumming signal, measured in impacts per second (Kettler and Leuthold 1995; Reinhard and Clément 2002; Barron and Plath 2017), varies between species (Howse 1964b; Table 1). Howse (1964b) initially hypothesized that the pulse repetition rate may be used to communicate the level of threat. However, he reported that the pulse repetition rate remained relatively constant, with the only factor changing it being the temperature. Howse (1964b) reported that the pulse repetition rate of *Z. angusticollis* linearly increases from 18 Hz at 17.5°C to 36 Hz at 23°C, above which the pulse repetition rate decreases with increasing temperature. Oberst et al. (2017) reported that the pulse repetition rate and amplitude of *Co. acinaciformis* alarms are like the footstep pattern of predatory ants *Ir. purpureus,* potentially indicative of evolutionary emergence and potential acoustic mimicry.

### 3.2 Vibration Sense Organs on Appendages

Insects use vibro‐mechanical sensing via chordotonal organs located throughout their bodies for vibration detection (Howse 1964b; Field and Matheson 1998; Takanashi et al. 2019; Yack et al. 2020). Chordotonal organs are stretched receptors at the cuticle and are also used to determine the joint position, i.e., the position at the leg or the antenna (Bässler 1988; Larsen et al. 1997; Yack 2004; Schmitz et al. 2019). Chordotonal organs of different functions consist of one to over one thousand scolopidia. Each scolopidium has up to two sensory neurons (Boo and Richards 1975; Strauß and Lakes‐Harlan 2017; Ishikawa et al. 2020), a glial (sheath) cell, and a cap cell to connect the neurons to the distal cuticle; a scolopale cell that surrounds the distal dendrite and supports mechanically the scolopidium's geometry; and a ligament cell that anchors the neurons to the proximal cuticle (Kavlie and Albert 2013).

**FIGURE 1:**
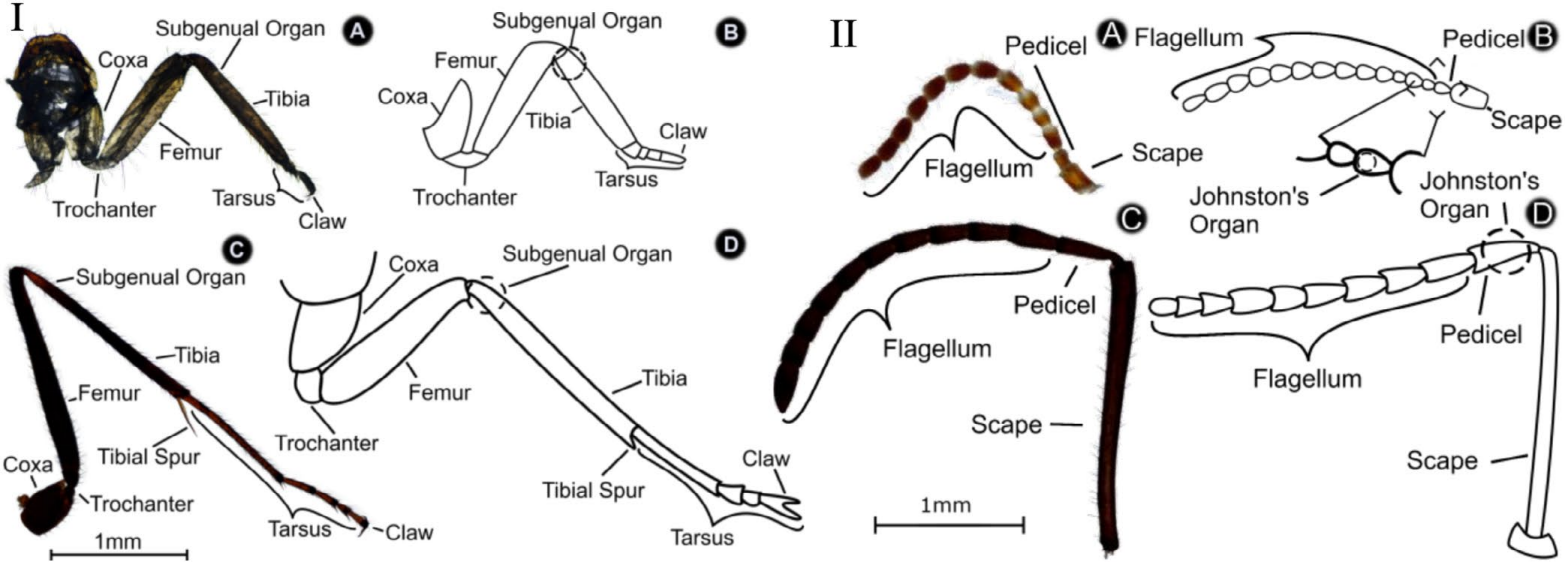
(I) Definition and comparison of the front legs of termites and ants. Image and schematic diagram of *Na. exitiosus* (soldier caste) front leg (A, B) in comparison with those of *Ir. purpureus* front leg (C, D), showing the components and locations of the subgenual organ (SGO) as the primary vibration‐sensing organ. (II) Definition and comparison of the antennae in termites and ants. Image and schematic diagram of *Na. exitiosus* (soldier) antenna (A, B) in comparison with the image and schematic diagram of *Ir. purpureus* antenna (C, D), showing Johnston's organ (JO).

The two major chordotonal organs that detect vibrations are SGO in the legs (Figure 1I) and JO located in the pedicles of the antenna (Figure 1II). The SGO is in the cuticle of the tibia, just below the femur, running across the haemolymph channel, and consists of multiple scolopidia (Demoll 1917; Howse 1964b, 1965b; Moran and Rowley 1975; Kilpinen and Storm 1997; Yack 2004). Scolopidia are sensory cells that are located across the haemolymph channel (Howse 1965b; Sansom et al. 2022). The antenna houses the JO, another organ known to be used by many other insects for vibration detection, which is in the first segment of the pedicel (Kirchner 1994; Yorozu et al. 2009; Mamiya et al. 2011; Ishikawa et al. 2020); see Figure 1II.

The leg and antennae of the termite *Na. exitiosus* (soldier) and the ant *I. purpureus* (worker) are schematized in Figure 1I,II. The leg of an insect consists of the coxa, trochanter, femur, tibia (with the SGO), tarsal segments, and claw. Compared with those of ants, the legs of termites are shorter and thinner relative to their body length, so the SGO is closer to the termite body. An antenna consists of the flagellum, the pedicel, and the scape (Figure 1II). In ants, the scape is much larger than that in termites, which moves the pedicel (with the JO) further away from the cuticle base at the head.

### 3.3 Legs and Antennae as Sensory Probes

The primary function of insect legs is locomotion. Leg morphology varies due to adaptations to locomotion type. Termites are social cockroaches, which are assumed to be ground runners and burrowers (Revzen et al. 2013), whereas ants, cf. Reinhardt et al. (2009) may be climbers on level surfaces. These may be complicating factors in understanding ant and termite legs as sensory probes for vibrational communication.

Setting aside surface contact conditions, the leg's main properties of interest include geometry (length, thickness, kinematics), and the material characteristics and dynamic properties (stiffness, damping) of the cuticular and attached tissue and joints. Vincent and Wegst (2004) examined literature on insect exoskeletons, including species from the following orders: Coleoptera, Diptera, Hemiptera, Hymenoptera, Lepidoptera, Orthoptera, and Phasmida. The general moduli of elasticity or stiffness of a variety of cuticle materials were approximated, ranging from 1 kPa for the soft cuticle to 150 GPa for the chitin nanofibers. Chen et al. (2013) reported that the stiffness of the wings of *Sympetrum flaveolum* due to the loss of haemolymph and general desiccation increased by 20.5 times for dead samples compared with 30 MPa in recently deceased (under 1 h) samples.

**FIGURE 2:**
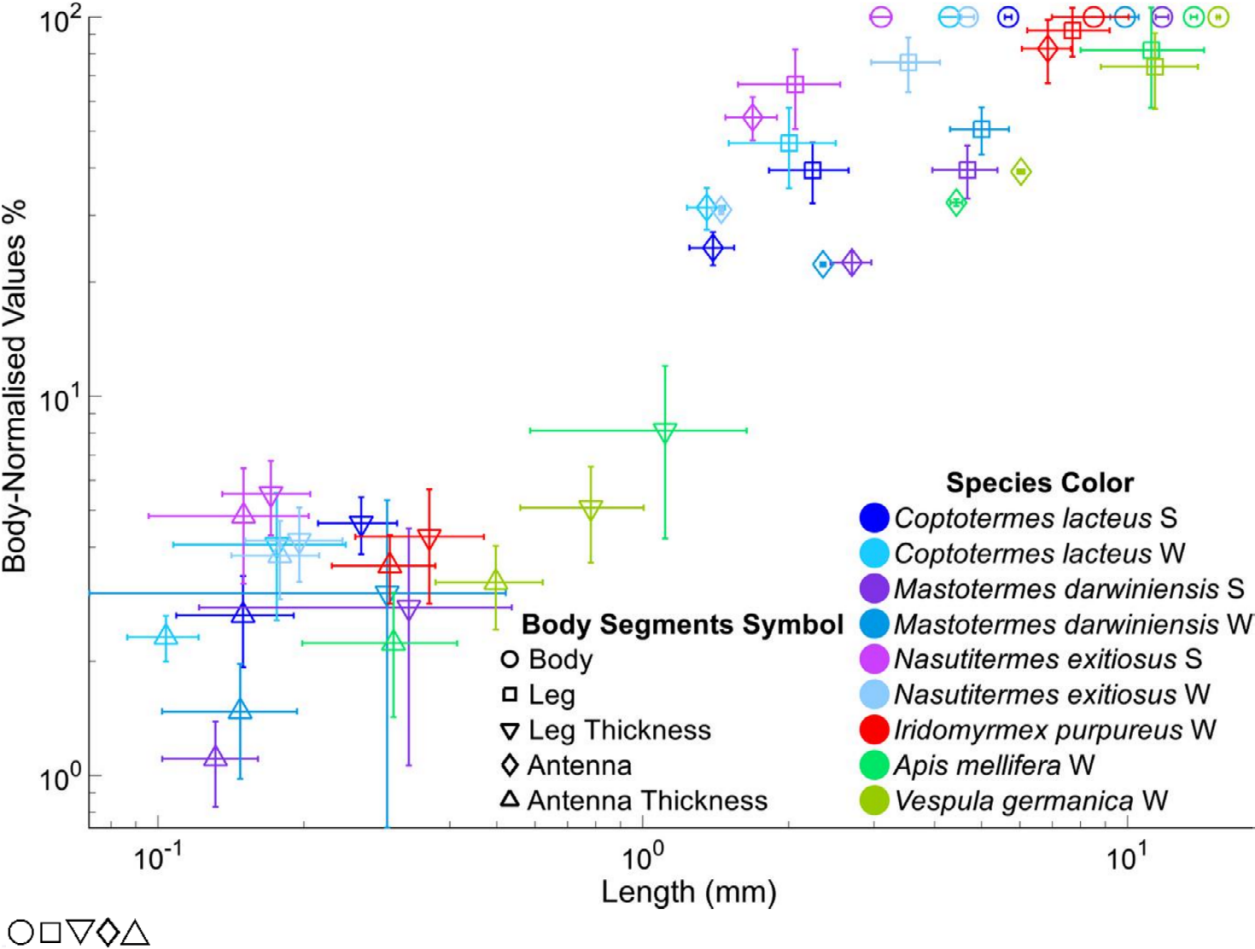
Graphical overview of body segment comparison of adults of social insect species. Body‐normalized values, presented in %, were determined by taking the individual segment lengths (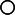 = Body, 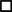 = Leg, 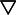 = Leg Thickness, 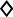 = Antenna, and 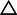 = Antenna Thickness) and then dividing by the body length for each specimen separately. The samples consist of *Co. lacteus* soldiers [S] (*n*_*S*_ = 7) and workers [W] (*n* = 9), *Na. exitiosus* (*n*_*S*_ = 9, *n* = 4), *Ma. darwiniensis* (*n*_*S*_ = 3, *n* = 4), *Ir. purpureus* (*n* = 6), *A. mellifera* , (*n* = 7), *V. germanica*, (*n* = 5).

The scatter plot in Figure 2 highlights morphological scaling differences among insect groups, with a broader representation of termite species compared to single representatives of ants ( *Iridomyrmex purpureus* ), bees ( *Apis mellifera* ), and wasps (*Vespula germanica*). Termites exhibit a wide range of body‐normalized values, particularly in leg and antenna proportions, which are, in general, shorter relative to body length compared to the species of the order Hymenoptera.

In the following, we studied the legs and antennae of termites and some selected Hymenopteran, especially the ant *Iridomyrmex purpureus* , an abundant predatory ant in Australia (Oberst et al. 2017). Figure 2 gives an illustrative overview of leg and antennae length relative to body size for some termite species and ants, as well as a wasp and a bee species.

Termites *Co. lacteus* and *Na. exitiosus* have absolutely and relatively shorter but thicker legs than the ant *Ir. purpureus*. The wasp (*V. germanica*) and the European honeybee ( *A. mellifera* ) have body‐normalized values of the antenna, and the leg length is like that of termites, while those of the ant studies are much larger. For all Hymenopteran, there is a larger standard deviation for the legs because of the variation in size between the front, mid, and hind legs, which is less pronounced in Isoptera.

Insect antennae generally have large numbers of sensilla or sensory hairs, which are used for detecting a range of stimuli, including olfactory, visual, touch, and taste stimuli (Yanagawa et al. 2009), along with other sensory organs, such as the JO. An antenna is manipulated using muscles attached at either the scape or pedicel and anchored to the head capsule (IMMS 1935; Ehmer and Gronenberg 1997) and varies in shape and function (Table 2).

**TABLE 2:** Comparison of legs and antennae between termites and ants.

Family species	Caste	Leg			Antenna			
		Length (mm)	Thickness (μm)	Slenderness ratio (*λ*)	Length (mm)	Thickness (μm)	Slenderness Ratio (*λ*)	Scape/Antenna (%)
Rhinotermitidae								
*Co. lacteus*	S	2.24 ± 0.42	263 ± 49	17.0 ± 8.6	1.40 ± 0.15	150 ± 41	18.9 ± 3.6	10 ± 2
	W	2.00 ± 0.50	176 ± 68	23.8 ± 4.6	1.36 ± 0.01	104 ± 17	26.0 ± 2.6	10 ± 2
Mastotermitidae								
*Ma. darwiniensis*	S	4.68 ± 0.72	329 ± 208	33.9 ± 30.7	2.70 ± 0.26	131 ± 29	41.2 ± 6.3	14 ± 1
	W	5.00 ± 0.69	297 ± 225	40.2 ± 34.5	2.35 ± 0.03	148 ± 46	32.8 ± 9.2	10 ± 1
Termitidae								
*Na. exitiosus*	S	2.06 ± 049	171 ± 35	24.2 ± 2.4	1.69 ± 0.20	150 ± 54	22.9 ± 7.2	7 ± 1
	W	3.53 ± 0.57	196 ± 44	36.1 ± 4.1	1.45 ± 0.02	178 ± 37	16.5 ± 3.3	8 ± 1
Formicidae								
*Ir. purpureus*	W	7.70 ± 1.49	363 ± 107	42.9 ± 10.2	6.85 ± 0.80	301 ± 72	46.2 ± 6.1	40 ± 8
Apoidea								
*A. mellifera*	W	11.2 ± 3.20	1112 ± 526	20.9 ± 10.3	4.44 ± 0.12	306 ± 108	30.0 ± 11.1	73 ± 11
Vespidae								
*V. germanica*	W	11.4 ± 2.58	782 ± 223	29.4 ± 2.6	6.03 ± 0.10	498 ± 124	24.8 ± 6.7	20 ± 5

*Note:* Leg and antenna length, thickness, slenderness ratio (*λ*), and percentage of antenna consisting of the scape of *Co. lacteus* soldier (S) *n* = 7; and workers (W) *n* = 9; *Ma. darwiniensis* (S) *n* = 3; workers (W) *n* = 4; *Na. exitiosus* (S) *n* = 9, (W) *n* = 4; *Ir. purpureus* (W) *n* = 6; *A. mellifera* (W) *n* = 7; *V. germanica* (W) *n* = 5. The thickness was determined at multiple points (3–10) across the length. Slenderness ratio calculated using “*λ* = *lr* ^−1^” where “*l*” is the length and “*r*” is the radius/half thickness (Oberst et al. 2018). Values refer to mean ± SD.

The termites have moniliform antennae (Figure 1IIA,B) and appear pearl necklace‐like (Triplehorn et al. 2005). The cockroach antennae take a setaceous form, which appears like a series of cylinders that taper out slightly at the end. Hymenoptera have geniculate antennae (Ward 2006; LaPolla et al. 2013), which consist of a large scape with its pedicel connected at right angles. The antenna characteristics of the species studied are summarized in Table 2. For *I. purpureus* and *A. mellifera* , the scape makes up approximately 30%–40% of the total antenna length, with this length slightly reduced for *V. germanica* at 20% of the total length. This is a major difference in physiology from Isoptera (also see Figures 1 and 2). Compared with ants, termites have a shorter antenna relative to body size; the termite *Na. exitiosus* workers had a shorter body‐normalized antenna length (30.6% ± 4.1%) than soldiers did (45.6% ± 4.2%). These values agree well with those reported by Castillo et al. (2021) for *Co. formosanus* soldiers (45.9% ± 0.5%) and workers (36.3% ± 0.6%). Ants have much longer antennae in comparison (76.0% ± 11.0%; Figure 2).

**FIGURE 3:**
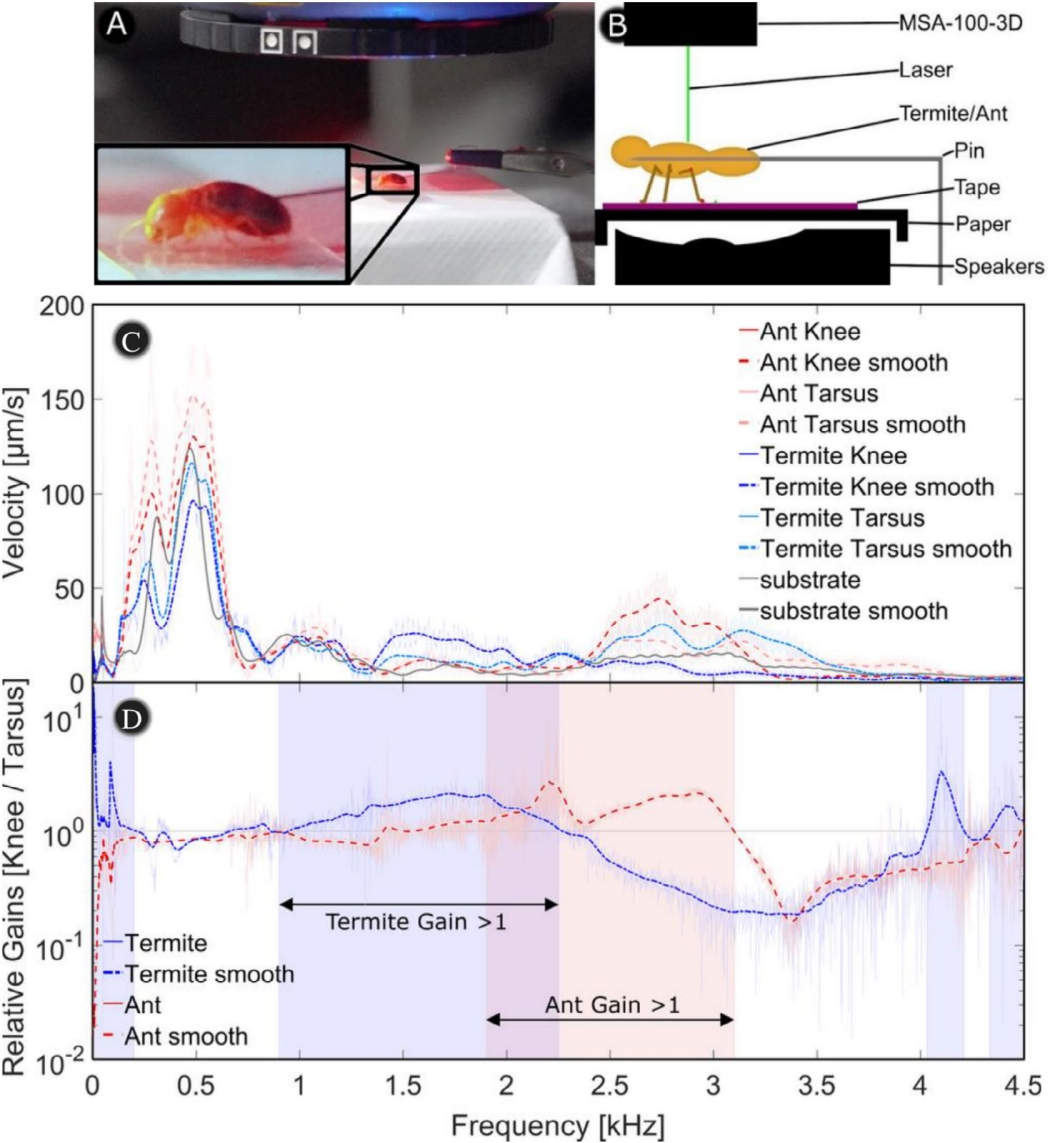
Experiments to conduct the vibration experiment. Microsystems analyser to measure leg vibration, pinned insect specimen in contact with paper substrate, and loudspeaker excitation. (A) setup, and (B) schematic. Vibration response and gain for termite (*Ma. darwiniensis*) and ant (*Ir. purpureus*). (C) Vibration response for tarsus and knee (tibia) with smoothed curves and (D) estimated gain by forming the ratio of knee vibration at the location of the SGO (cf. Figure 1) response to tarsus.

A range of factors can determine the capability of the leg, and the associated vibrations transmitted to the SGO, and the question is: How do ant and termite legs differ to allow termites to sense vibrations differently from predatory ants? How legs and the antenna, their length and geometry, contribute to vibration sensing is yet to be explored. While little is known about the function of the JO in termites, the SGO has been studied more reliably as a vibration‐sensing organ. To explore how the leg as the sensory probe contributes potentially to vibration sensing of the SGO, we studied the vibration response at different locations in the termite leg with focus on the transmission from the termite foot, in contact with a substrate, to the tibia where the SGO is located (Figure 1; cf. Sansom et al. 2022). The results of this analysis are shown in Figure 3.

We conducted vibration measurements using a Polytec MSA100‐3D Micro System Analyser to record the response spectra of ant and termite legs under random vibration excitation. These responses were then related to morphological features, particularly appendage length and thickness relative to overall body size. To avoid tissue damage, the MSA laser intensity was maintained at 30% power (~250 μW), following the protocol of Sansom et al. (2022). The resulting signals were smoothed using a moving average filter with a window of 100 samples (25 Hz; see Data S3 and S5).

To estimate the gain, we calculated the ratio of vibration amplitudes measured at the tarsus (foot, input location) to those at the tibial subgenual organ (SGO, knee, output location) for the left front leg of both a termite and an ant. The experimental setup is illustrated in Figure 3. A freshly prepared *Ma. darwiniensis* specimen (less than 60 days old) was placed in ethanol and then pinned through the head capsule on a fine needle for more than 30 min to allow residual ethanol to evaporate. Measurements were conducted within the subsequent 30 min to minimize dehydration of the specimen. Pinning through the head capsule was selected as it provided the most structurally robust anchoring point to reliably hold the termite during measurement.

The pinned specimen was positioned in a near‐natural posture with its legs resting on a stretched sheet of tissue paper secured with adhesive tape over a loudspeaker. The loudspeaker provided substrate vibrations that excited the legs, while their responses were measured with the microsystem analyser. The MSA system was operated with a working stand‐off distance of 38 mm and a depth of focus of ±10 μm to ensure accurate capture of vibrational motion.

In Figure 3C, four raw measurements and averaged/smoothed (Data S4) measurements are presented. While the data are very noisy, due to the sensitive structure of the legs studied, the averaged curves show distinct peaks. In Figure 3C, the gain estimates and the termite sample seem to be able to transfer vibrational information in the range from about 0 Hz to about 200 Hz and 900 Hz to about 2.25 kHz, while the ant leg shows a quite different frequency range from about 1.9 kHz to 3.1 kHz.

In Figure 3C, four raw measurements and averaged/smoothed (Data S4) measurements are presented. While the data are very noisy due to the sensitive structure of the legs studied, the averaged curves show distinct peaks. In Figure 3C, the gain estimates and the termite sample seem to be able to transfer vibrational information in the range from about 0 Hz to about 200 Hz and from 900 Hz to about 2.25 kHz, while the ant leg shows a quite different frequency range from about 1.9 kHz to 3.1 kHz.

### 3.4 Internal Vibration Sensors SGO and JO

SGOs vary among all insect species in terms of size, shape, and number of scolopidia (of *Z. angusticollis* ). Their club‐shaped SGOs (Howse 1965b) are assumed to be most sensitive to frequencies of up to 6 kHz (Howse 1964b; Kirchner et al. 1994), whereas cockroaches (Blattidae) have fan‐shaped or club‐like SGOs (Moran and Rowley 1975), depending on the species (Table 3). However, vibration sensitivity, the frequency range for Blaberidae, and the number of scolopidia for Archotermopsidae and Blattidae, as well as the role of shape, are unknown (Table 3).

**TABLE 3:** Substrate sensing organs, their frequency range, SGO shape, angle relative to the haemolymph channel, and number of scolopidia.

Family species	Frequencies (kHz)	Shape	Angle (°)	Scolopidia #	References
Hymenoptera					
Apidae					
*Apis mellifera*	0.15–0.9	Hollow cone	~10	~40	Kilpinen and Storm 1997; Sansom et al. 2022
Formicidae					
*Camponotus ligniperda*	—	Deformed sphere	~1	35–40	Menzel and Tautz 1994; Sansom et al. 2022
*Ir. purpureus*	—	—	~6	—	
Blattodea					
Blaberidae					
*Blaberus discoidalis*	—	Fan	—	40–50	Meusemann et al. 2020; Moran and Rowley 1975
*Blattella germanica*	—	Fan	—	40–50	Meusemann et al. 2020; Moran and Rowley 1975
Blattidae					
*Periplaneta americana*	< 5	Club	—	—	Bourguignon et al. (2015); Meusemann et al. (2020); Schnorbus (1971)
Mastotermitidae					
*Mastotermes darwiniensis* S	—	—	~26	—	Data S7
*Mastotermes darwiniensis* W	—	—	~28	—	Data S7
*Termitidae*					
*Na. exitiosus*	—	—	~19	—	Sansom et al. 2022
*Z. angusticollis*	< 6	Club	—	—	Bourguignon et al. (2015); Meusemann et al. (2020); Howse (1964b)
Austrophasmatidae					
*Karoophasma biedouwense*	0.3–1.3	Fan	~1	15–30	Eberhard et al. (2010); Sansom et al. (2022)

*Note:* Cells; ‘—’ = no information identified, ‘~’ = value has been predicted based on existing μCT‐scan data (ref. Sansom et al. (2022) for more information).

Howse (1964b) proposed that the SGO of the termite *Z. angusticollis* uses two types of cells distributed throughout the SGO. If the termite is exposed to a vibration stimulus, the two cells are set in motion, generating transient oscillations that can then be detected. The SGO is more sensitive to vertical oscillations than horizontal oscillations (Kilpinen and Storm 1997; Strauß and Lakes‐Harlan 2017; Strauß et al. 2019). This is attributed to the interactions between the SGO, the residual haemolymph channel and the haemolymph itself, with the haemolymph being used to transmit vibrations in the form of pressure waves detected by the SGO. This interaction with the SGO works best when the pressure waves are pushing against the SGO (vertical oscillations) rather than running across the SGO (horizontal oscillations).

The angle of insertion of the SGP in the leg may also be important. The SGO in termites has a relatively large angle of 19.2° to the haemolymph channel, which is larger than those of the other insects investigated (Table 3). This larger angle may facilitate vibration detection, particularly that of horizontal vibrations (Sansom et al. 2022). By having an SGO in each leg, the termite *Macrotermes natalensis* soldiers and workers can take advantage of the slight time delay (as small as 0.2 ms) between their legs to determine the direction of the signal (Hager and Kirchner 2014; Hager et al. 2019). The situation may be different for ants. Hager et al. (2017) showed that the body size of ants is sufficient for a vibrotropotactic orientation based on the analysis of time‐of‐arrival delays and that the ant ( *Atta sexdens* ) uses time‐of‐arrival delays smaller than 0.3 ms.

Jerking signals in termites (Hager et al. 2019) directly impact the antenna (Howse 1965a; Sim and Lee 2013, 2017; Hertel et al. 2011), along with producing transverse waves, for which the SGO is likely to be less effective at detection. The SGO is positioned across the haemolymph channel, with this fluid‐filled chamber used to help transmit vibrations to the SGO, where past studies on the honeybee *A. mellifera* and the stick insect *Sipyloidea sipylus* have shown it to be less sensitive to longitudinal waves (Kilpinen and Storm 1997; Strauß and Lakes‐Harlan 2017; Strauß et al. 2019).

The JO may be used to detect airborne or substrate‐borne vibration signals. The honeybee *A. mellifera* and vinegar fly *Drosophila melanogaster* appear to use their JOs to detect airborne sounds (Kirchner 1994; Ai et al. 2009; Yorozu et al. 2009; Ishikawa et al. 2020), whereas other insects, including *A. mellifera* and the ant *Camponotus vagus*, seem to use them to detect substrate‐borne vibrations (Kirchner 1994; Hunt and Richard 2013). The antennae of termites possess a variety of sensory organs, including a JO (Figure 1IC), and sensilla or sensory hairs, which are used for detecting stimuli, including semiochemicals, touch, and temperature (Yanagawa et al. 2009). The JOs of termites are in the pedicel directly adjacent to the first flagellum segment, which is the smallest antennal segment in termites; see Figure 1II (Snyder 1926; Gay 1974; Yanagawa et al. 2009; Hunt and Richard 2013; Fu et al. 2020).

The small size of the pedicel and positioning of the JO thereon may be important in the detection of vibration signals in termites. The small size may help to amplify the vibrations across the antenna while shifting their resonance frequencies. This may also improve the antenna's function as a sensory probe for the JO; however, no research has been conducted on this topic to date. The slenderness ratio (*λ*) of the antenna in termites ranges, for the species tested, in workers from 10.9 to 34.5 (*Na. exitiosus*) and in soldiers from 25.1 to 37.3 (*Na. exitiosus*) and 18.6 to 35.8 (*Co. lacteus*; Table 2). This is far smaller than for the other insects investigated, ranging from 22.5 to 76.8 (Table 2). Larger values of *λ* are related to greater modal density, resulting in lower natural frequencies, and lower *λ* values result in both more discrete and higher modal frequencies (Oberst and Tuttle 2018). However, more research on antennae as vibration sensors is necessary to compare different insect genera and to ascertain whether broad sensitivity (related to a higher density of modes) or higher sensitivity to specific frequencies (rather than discrete vibration mode detection) is preferred.

## 4 Discussion

Compared with crickets, which belong to the order Orthoptera, one of the best‐studied insect orders concerning vibro‐acoustic communication (Stritih and Strauß 2018; Velilla et al. 2020; Brandt et al. 2023), and for which the subgenual organ complex has been studied in detail (Strauß and Lakes‐Harlan 2017), termites exhibit a narrower and lower frequency sensitivity range. Crickets use tympanal organs resonating between 1 kHz and 20 kHz, with some species capable of ultrasonic detection up to 40–100 kHz, while their mechanosensory transmission typically remains below 5 kHz, often in the sub‐kHz range. Cave crickets, which are specialized in vibrational communication and lack acoustic hearing, are most sensitive to vibrations from 10 Hz to 120 Hz, occasionally up to 500 Hz (Stritih and Čokl 2012). Their legs function as bandpass filters shaped by material properties and kinematics (Stritih and Strauß 2018). By contrast, termites show lower‐frequency specialisation, reflecting adaptations to their substrate‐borne communication channels. Crickets often sit on leafy matter, with a preference for leaves and shoots, but can be omnivorous, so substrates may change.

### 4.1 Morphology and Sensory Specialisation in Termites and Ants

Despite functional similarities in vibrational sensing, the sensory ecology of termites and ants remains comparatively understudied. Little research on termites is concerned with the subgenual organ (SGO), while leg shape, material composition, and proportion scaling have been overlooked. Like crickets, we may consider the SGO to be less sensitive to longitudinal waves (Strauß et al. 2019). However, haemolymph pressure and oscillation direction can alter the mechanical input reaching the SGO, modulating its sensitivity (Kilpinen and Storm 1997; Strauß and Lakes‐Harlan 2017). By contrast, the antenna and Johnston's organ (JO), which directly contact jerking individuals (Howse 1965a; Hertel et al. 2011; Sim and Lee 2013, 2017), may be better suited for detecting in‐plane oscillations that bypass SGO insensitivity. In this context, in *Nasutitermes exitiosus*, a constriction of haemolymph channels around the SGO has been identified, which may amplify vibration components (Sansom et al. 2022), with an SGO's 19.2° orientation relative to the substrate that may facilitate conversion of in‐plane forces into detectable signals, thus supporting otherwise even harder‐to‐detect jerking vibrations which are often in‐plane.

We found that termite legs and antennae are shorter than those of Hymenopterans (Figure 1), yet both groups produce similar signal time‐of‐arrival delays useful for tropotactic orientation (Hager and Kirchner 2014; Hager et al. 2017), which is probably due to the substrate properties. However, termites of *Macrotermes* showed time/phase‐based directional sensing, while ants of *Atta* showed amplitude‐based directional sensing, a divergence that suggests substrate‐vibration communication has evolved different sensory coding strategies in social insects. This difference may reflect different ecological pressures since termites live in dark, enclosed environments (where phase cues are stable due to similar tunnel shape and clay structures), versus ants foraging in heterogeneous soils and vegetation (where amplitude cues may be more reliable). Phase cues for directional vibration sensing are more reliable in the subterranean and enclosed environments occupied by termites than in the heterogeneous substrates used by ants. In termite galleries, vibrations propagate through relatively homogeneous materials such as soil, carton, or wood, where wavefronts remain coherent over short distances (Oberst, Lai, and Evans 2019; Oberst, Lenz, et al. 2019). This allows termites to exploit minute inter‐leg arrival‐time differences with high temporal precision. The enclosed setting also shields vibrations from environmental noise and reduces interference from external sources such as wind or air currents, thereby preserving the phase structure of the signal (Oberst et al. 2020). Moreover, while amplitude can fluctuate considerably depending on soil compaction, contact quality, or structural irregularities, the relative timing of wave arrival is less sensitive to such variation, making phase a more stable cue underground. By contrast, leafcutter ants forage in open environments where vibrational signals are transmitted across diverse and irregular substrates such as leaves, twigs, and soil surfaces. In these settings, signals are subject to scattering, reflection, and refraction, which rapidly degrade temporal quality. Under such conditions, amplitude gradients between legs provide a higher consistent directional indicator than a phase angle. It might therefore be argued that ecological context has driven termites and ants to evolve distinct coding strategies for extracting directional information from substrate vibrations.

The more compliant legs of termites may increase modal density at lower frequencies, thereby enhancing sensitivity. This may enable termites to detect low‐frequency signals, such as predator cues, more efficiently, since such waves travel rapidly from the ground to the SGO. Shorter legs may improve manoeuvrability on smooth nest surfaces (Oberst et al. 2020) and stability, whereas ants navigate more cluttered external environments (Hartmann et al. 2020). Ultimately, the location and orientation of both the SGO in the leg and the JO in the antenna are likely to influence signal detection, shaping sensitivity, and specialisation: direct ground contact through the legs promotes narrowband detection, while antennae support complementary in‐plane sensitivity. Yet, very little research has been conducted in this regard.

### 4.2 Antennae and Johnston's Organ

Antenna shapes, including their slenderness ratios, provide little obvious explanation for interspecific differences between ants and termites (Figure 2; Table 2). However, comparing termites, workers show a larger slenderness ratio than soldiers, which mechanically implies higher modal density at lower frequency and potentially more resonances, increasing vibration sensitivity, like what was found for the legs. Yet, antennae are also critical for semiochemical communication; thus, length may simply accommodate more sensory hairs or greater surface coverage important to sense better, more chemicals. The JO, located in the pedicel, has been previously characterized in other insects such as honeybees and flies for detecting airborne and substrate‐borne vibrations (Ai et al. 2009; Yorozu et al. 2009), but in termites, its function remains speculative. Its small size and proximity to the head may enhance in‐plane jerking sensitivity, complementing the SGO, which is less suited to such oscillations. Structurally, the JO in termites is formed as a pearl‐like sequence of discrete masses, which could tune natural frequencies and localize vibration modes, akin to phononic band gap effects where mass arrangements suppress or channel wave propagation (Jia et al. 2018). Thus, antenna morphology, slenderness ratio, and scape proportions may contribute to complementary frequency sensitivities between the SGO and JO.

### 4.3 Preliminary Experimental Results

We present the first detailed examination of termite (*Mastotermes darwiniensis*) and ant ( *Iridomyrmex purpureus* ) leg structures using a Polytec microsystems analyser. *M. darwiniensis* was selected for its relatively large size and handling suitability. Although *Ir. sanguineus*, a close relative of *Ir. purpureus*, is more abundant in the Northern Territory (NT), the overlapping ranges of *M. darwiniensis* and *Ir. purpureus* suggest natural encounters are still likely. We therefore measured vibration transmission from the tarsus to the SGO and observed frequency‐dependent gain amplification in termite legs below 120 Hz and between 900 and 2.25 kHz, resembling patterns in cave crickets (Stritih and Čokl 2012; Stritih and Strauß 2018). Termite legs also displayed an attenuation zone from 2.2 kHz to 4 kHz, which could mean that they are adapted to wood and clay substrates, which are often found in lower frequency ranges, near 0 Hz and up to 2.25 kHz. Similar to MEMS resonators, the legs could therefore be tuned to specific eigenfrequencies (Dienel et al. 2012; Maeda et al. 2006). In contrast to *M. darwiniensis*, *Ir. purpureus* legs exhibited sharp peaks at 2.18 and 2.95 kHz (1.9–3.1 kHz range), with gain mostly above 2 kHz. These findings suggest distinct vibrational specialisations but leave open the question of how vibrational energy is efficiently transferred from substrate to leg (Oberst et al. 2020).

### 4.4 Predator–Prey Sensory Interactions

Ants are opportunistic predators and frequently nest close to or within termite mounds (Oberst et al. 2017), preying on termites. Ant–termite interactions thus represent an important but underexplored ecological linkage (Tuma et al. 2020). The partial overlap in vibrational frequency sensitivity between *I. purpureus* and *M. darwiniensis* resembles predator–prey systems such as cats and mice (Heffner and Heffner 1988), suggesting possible co‐evolutionary sensory adaptations. However, frequency overlap alone is insufficient evidence; direct proof of reciprocal selective pressures is required. Enhanced low‐frequency sensitivity in termites may represent an adaptive strategy for communication through channels less susceptible to predator interception. As ants rely heavily on chemical signaling while termites exploit vibrational cues, multimodal divergence may further reduce eavesdropping. Nevertheless, the persistence of partial overlap maintains evolutionary pressure for divergence and fine‐tuning of communication channels.

### 4.5 Limitations and Methodological Considerations

However, the mechanical roles of legs and antennae in vibration transmission remain poorly understood. Signal amplitudes were very small and prone to noise, particularly at high frequencies (see Data S6), and thus must be interpreted cautiously. More samples per species and alternative excitation methods are needed to validate our findings. Non‐normal acoustic excitation and thermal excitation (e.g., Brownian motion, thermomechanical noise), widely used in designing MEMS, represent promising options (Ruppert et al. 2025). Additionally, substrate effects, transmission, reflection, and coupling via feet could be considered (Büscher et al. 2018; Brandt et al. 2023). High‐resolution 3D μCT scans, combined with stiffness and dynamic modelling (Spinner et al. 1960; Quinn and Swab 2004; Platz and Schmid 2019; Pedersen 2000) could facilitate model updating and interesting sensitivity studies. Given that we measured only six species (three termites, one ant), a far greater diversity is needed across the ~3200 termite and ~15,000 ant species to uncover a broader range of patterns (Wilson 1968; Oberst et al. 2020).

### 4.6 Concluding Remarks and Outlook

Future research should treat the leg as an integrated vibro‐acoustic sensing complex and examine the JO's frequency response and neural integration, using electrophysiology or μCT‐based biomechanical modelling, and compare antennal structures across species to assess variation in vibrational sensitivity. While the SGO is regarded as the primary vibration‐sensing organ (Strauß et al. 2024), accumulating evidence suggests the JO and antennae play complementary roles (Roces and Tautz 2001; Kumari et al. 2006). The SGO primarily detects out‐of‐plane vibrations, while jerking signals occur in‐plane (Hertel et al. 2011; Hager et al. 2019). Dual sensors with distinct sensitivities, narrowband versus broadband, in‐plane versus out‐of‐plane, near‐field versus far‐field, may enhance signal‐to‐noise ratios, like sensor fusion in engineered systems (Madgwick et al. 2013). Thus, legs and antennae act as probes, with the SGO and JO as complementary transducers, together forming a distributed vibration detection apparatus analogous to the human ear (Gale et al. 2004; Minckler et al. 1977). This integrated perspective highlights the role of vibrational sensing in termite self‐organized architectures (Oberst and Martin 2024) and communication networks critical for colony survival.

Vibrational signals are essential in termite alarm signalling, foraging coordination, and nestmate recognition (Hager and Kirchner 2014; Oberst et al. 2017). Dual use of SGO and JO may allow multimodal detection across near‐ and far‐field cues, supporting complex social behaviours and architectural decision‐making. Future work should therefore explore how vibrational sensing integrates with stigmergic processes in self‐organisation (Oberst et al. 2020), potentially linking vibration input to behavioural algorithms not yet associated with vibrational cues (Werfel et al. 2014; Huang et al. 2025).

Vibration‐sensing strategies are likely to be species‐specific and ecologically shaped. Termites often forage in acoustically damp environments (Oberst, Lai, and Evans 2019), where low‐frequency vibrations propagate efficiently (Mortimer 2017). Their short, compliant legs may be tuned for such conditions, while ants in heterogeneous terrains may favour higher‐frequency detection. Termite physiology and sensory abilities may further differ among wood‐eating, grass‐harvesting, and soil‐feeding species, including those cohabiting with wood‐dwellers. Likewise, comparisons among ants, seed harvesters, carnivores, and termite specialists may reveal sensitivity differences linked to ecological roles. Bridging these physiological traits with environmental context remains an outstanding challenge, but also a promising avenue for understanding how termites communicate and shape their environments (Perna and Théraulaz 2017). In this context, it might be crucial to link the mechanics of especially termite sensory organs (complexes) with the physics of wave propagation in substrates they build to walk and live in as a communication channel, which remains poorly explored (Oberst et al. 2020).

## Author Contributions

**Travers M. Sansom:** conceptualization (equal), data curation (equal), formal analysis (equal), investigation (equal), methodology (equal), validation (equal), visualization (equal), writing – original draft (equal), writing – review and editing (equal). **Joseph C. S. Lai:** conceptualization (equal), writing – review and editing (equal). **Benjamin J. Halkon:** supervision (equal), validation (equal), writing – review and editing (equal). **Theodore A. Evans:** conceptualization (equal), writing – review and editing (equal). **Sebastian Oberst:** conceptualization (equal), funding acquisition (equal), methodology (equal), project administration (equal), resources (equal), supervision (equal), validation (equal), writing – original draft (equal), writing – review and editing (equal).

## Conflicts of Interest

The authors declare no conflicts of interest.

## Supporting information


**Data S1:** ece372287‐sup‐0001‐supinfo.docx.

## Data Availability

Data is provided in Data S1 and data depository https://doi.org/10.5281/zenodo.14997318.
