# Supplementary material for: Termite Vibration Sensing: The Chordotonal Organs and Their Appendages
Source: Ecol Evol. 2025 Oct 17;15(10):e72287. doi: 10.1002/ece3.72287 (PMC12531595; doi:10.1002/ece3.72287)
Supplement: Supplementary file 1 — Data S1: ece372287‐sup‐0001‐supinfo.docx. [file ECE3-15-e72287-s001.docx]

####

# Supplementary Material

Termite vibration sensing: the chordotonal organs and their appendages

Travers M. Sansom^a,*^, Joseph C.S. Lai^b^, Benjamin J. Halkon^a^, Theodore A. Evans^c^, Sebastian Oberst^a*^

^a^Centre for Audio, Acoustics and Vibration, University of Technology Sydney, Sydney, NSW 2007, Australia

^b^School of Engineering and Technology, University of New South Wales Canberra, Northcott Dr, Campbell, ACT 2612, Australia

^c^School of Biological Sciences, The University of Western Australia, Crawley, WA 6009, Australia

*Address correspondence to [travers.m.sansom@student.uts.edu.au](mailto:travers.m.sansom@student.uts.edu.au) or [sebastian.oberst@uts.edu.au](mailto:sebastian.oberst@uts.edu.au)

## S1 Sample procurement and storage

*Coptotermes lacteus*, *Nasutitermes exitiosus*, *Iridomyrmex purpureus* and *Polyrhachis ammon* on the 11 Nov 2019 for which the authors acknowledge a licence for scientific activities under the Nature Conservation Act 2014 within the ACT Tidbinbilla Nature Reserve (S.O. licence TS20188).

Samples were stored in small test tubes in ethanol, separated by species and colony. Samples were placed in the test tubes shortly after capture.

## S2 Measurement technique

Samples were first removed from test tubes and placed on a paper towel to remove any excess ethanol from the samples. The samples were then placed on a microscope slide under a camera. The microscope slides were placed at the same location for the photograph, with the sides of the microscope slide used as reference dimensions. Each photo was then imported into the image processing program Gimp v2.10.20 where the exact pixel coordinates of both the edges of the microscope slide and body sections were determined. Based on these, the length of the legs, antenna, and body was determined.

The legs and antennae were measured as the total length of all segments, with the leg including the total length of the coxa, trochanter, femur, tibia, and tarsus, and the antenna being the combined length of the scape, pedicel, and flagellum segments. Legs and antennae were only measured if the start and the end point of each segment were visible, with specimens imaged from multiple positions to allow for measuring more of each individual’s legs and antennae and assisting in improving the accuracy of the results.

The body length was similarly measured with the total being a combination of the distance between the base of the mandibles and the back of the head plus the total length of the alitrunk and petiole, and the base of the gaster to the end of the gaster for species without a stinger, or to the base of stinger if the species possessed a stinger.

Based on all of these values, the average lengths of each individual’s legs, antennae, and body length were determined as shown in Table S1. These values were then used to determine the average body length and body-normalised leg and antenna length of each species, as shown in Table 2.

## S3 Table

Table showing measured lengths of legs, antennae and body length for termites, ants, bees, and wasps. ‘S’ denotes soldier, ‘W’ denotes worker, ‘NA’ denotes not applicable.

| Family  Species | Caste | Body length (mm) | Body-normalised data (%) | | | N |
| --- | --- | --- | --- | --- | --- | --- |
|  |  |  | leg length | antenna length | antenna thickness |  |
| Rhinotermitidae |  |  |  |  |  |  |
| *Cop. lacteus* | S | 5.08 ± 0.06 | 35.2 ± 5.1 | 24.7 ± 1.7 | 2 ± 0.6 | 7 |
| Termitidae |  |  |  |  |  |  |
| *Na. exitiosus* | S | 3.11 ± 0.09 | 57.5 ± 11 | 45.6 ± 4.2 | 3.3 ± 1 | 9 |
|  | W | 4.68 ± 0.12 | 53.7 ± 15.3 | 30.6 ± 4.1 | 2.9 ± 0.7 | 4 |
| Formicidae |  |  |  |  |  |  |
| *Ir. purpureus* | S | 5.47 ± 0.41 | 84.4 ± 15 | 61.2 ± 8.7 | 2.9 ± 0.8 | 7 |
| *Polyrhachis ammon* | S | 7.7 ± 0.99 | 83.5 ± 4.7 | 75.8 ± 8.7 | 2.6 ± 0.5 | 6 |
| Apoidea |  |  |  |  |  |  |
| *A. mellifera* | W | 13.45 ± 0.15 | 60.1 ± 18.8 | 32 ± 0.5 | 1.5 ± 0.5 | 7 |
| Vespidae |  |  |  |  |  |  |
| *V. germanica* | W | 14.37 ± 0.07 | 61 ± 14.5 | 41.2 ± 0.2 | 2.5 ± 0.7 | 5 |

## S4 Matlab code for averaging / smoothed

It was found that “N=250” would give relatively smooth results while still retaining most detail

function V=average(inV,N)

x=50;

N=N*x;

for j=1:(size(inV,1))

in(j,:)=interp1(1:size(inV,2),inV(j,:),1:1/x:size(inV,2),'spline');

end

M=1:round(N/2);

M(round(N/2)+1:N)=rot90(rot90(M(1:round((N-1)/2))));

M=M.^5;

for i=min(size(in,2)-N,N):-1:1

T=M(1:i)/sum(M(1:i));

for j=1:(size(in,1))

V(j,i)=sum(in(j,1:i).*T);

V(j,size(in,2)-i)=sum(in(j,size(in,2)-i+1:size(in,2)).*T);

end

end

if size(in,2)-N>N

T=M/sum(M);

for j=1:(size(in,1))

for i=N+1:(size(in,2)-N)

V(j,i)=sum(in(j,i-N:i-1).*T);

end

end

end

end

## S5 Vibration response and gain for termite and ant - larger frequency interval

###
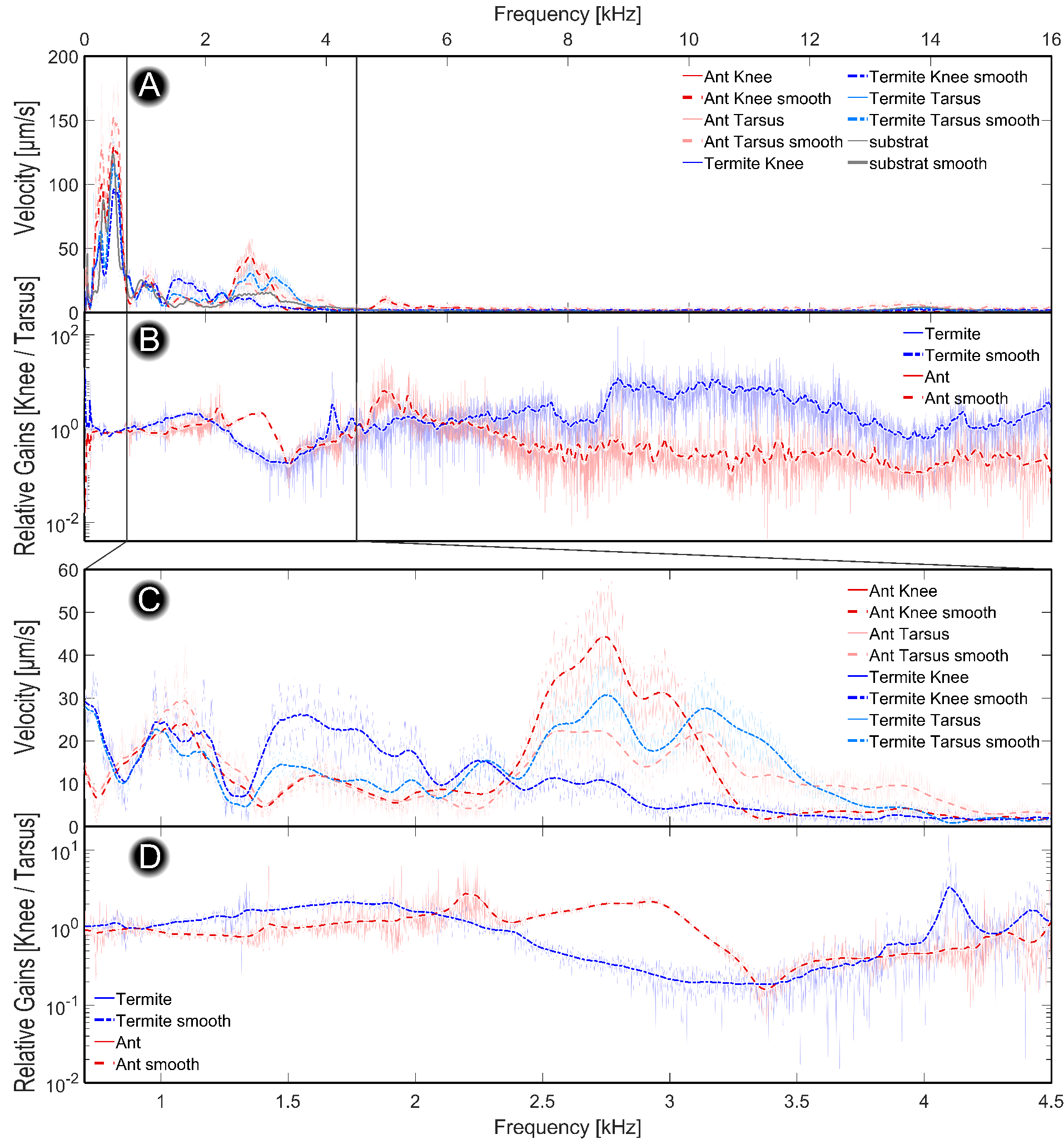


### Figure S1. Vibration response and gain for termite (*Ma. darwiniensis*) and ant (*Ir. purpureus*). (A,C) Vibration response for tarsus and knee (tibia) with smoothed curves and (B,D) estimated gain by forming the ratio of knee vibration at the location of the SGO (cf. Fig. 2) response to tarsus

## Figure S1 shows the vibration response of the termite and ant leg and foot, along with their ratio, where the tarsus is used as the input point and the vibration is measured at the tibia, where the SGO is located, to obtain an estimate of gain. As opposed to Fig. 3 (see main document), the higher frequency also shows a larger gain for the termite, which could indicate that termites are also more sensitive at the higher frequency range. However, since the quantities measured, especially in this upper frequency band, are so small and the measurements are noisier, results need to be interpreted with care, and more validating experiments are required to confirm findings in this paper.

##

## S6 Additional CT scans of *Mastotermes darwiniensis* SGO

Mastotermes darwiniensis S/W and Ir. purpureus SGO

Doi: 10.5281/zenodo.14997318
